# THR-β Agonists vs Incretin Therapies for Noncirrhotic Metabolic Dysfunction-Associated Steatohepatitis (MASH): A Biopsy-Anchored Systematic Review With Grading of Recommendations Assessment, Development and Evaluation (GRADE) Certainty

**DOI:** 10.7759/cureus.106038

**Published:** 2026-03-28

**Authors:** Amirah A Alzaki, Mohammed Z Alqahtani, Eiman Aman, Ghala M Asiri, Mada H Al Jibril, Rodan Desoky, Saad A Alqahtani, Lujain Suhaqi, Sarah A Alkhalid, Dai Y Alshareef, Mahmoud S Desoky

**Affiliations:** 1 Medicine and Surgery, University College Dublin, Dublin, IRL; 2 Medical School, King Khalid University, Abha, SAU; 3 Medical School, King Abdulaziz University, Jeddah, SAU; 4 Medical School, College of Medicine Alfaisal University, Riyadh, SAU; 5 Medical School, Jazan University, Jazan, SAU; 6 Gastroenterology, Sultan Bin Abdulaziz Humanitarian City, Riyadh, SAU

**Keywords:** efficacy of resmetirom, glp-1 receptor agonists, liver fibrosis, metabolic dysfunction-associate, non-alcoholic fatty liver disease, randomized controlled trials, resmetirom

## Abstract

Metabolic dysfunction-associated steatohepatitis (MASH) is a progressive liver disease now targeted by new pharmacotherapies, including the thyroid hormone receptor beta (THR-β) agonist resmetirom, glucagon-like peptide-1 (GLP-1) receptor agonists (semaglutide, liraglutide), and the dual glucose-dependent insulinotropic polypeptide (GIP)/GLP-1 receptor agonist tirzepatide. Each acts through distinct metabolic pathways, yet no head-to-head comparisons exist. The aim of this study was to systematically review randomized controlled trials assessing the efficacy and safety of resmetirom, GLP-1 receptor agonists, and dual GIP/GLP-1 receptor agonists in adults with biopsy-confirmed, non-cirrhotic MASH (F2-F3), and to evaluate the certainty of evidence using Grading of Recommendations Assessment, Development and Evaluation (GRADE). Following Preferred Reporting Items for Systematic Reviews and Meta-Analyses (PRISMA) 2020, seven eligible randomized controlled trials (RCTs) involving 3,796 participants were identified through PubMed, Embase, Cochrane Central Register of Controlled Trials (CENTRAL), and trial registries (inception to October 2025). The comprehensive methodological protocol was filed in PROSPERO (reg. no. CRD420251230032) before the commencement of data collection. Data were extracted in duplicate; risk of bias was assessed using Cochrane Risk of Bias 2 (RoB 2), and certainty of evidence was graded using GRADE. Due to heterogeneity, the data were synthesized qualitatively. The included studies were RCTs of adults with biopsy-confirmed, non-cirrhotic MASH (F2-F3) treated with resmetirom, GLP-1 receptor agonists, or GIP/GLP-1 receptor agonists, while non-randomized studies and pediatric and cirrhotic populations were excluded. This study is limited by the absence of head-to-head trials comparing the three drug classes, as well as by heterogeneity across the included studies, which precluded meta-analysis. The results showed that all active therapies outperformed placebo on at least one biopsy-anchored endpoint. Resmetirom improved both MASH resolution and fibrosis at 52 weeks, with marked low-density lipoprotein cholesterol (LDL-C) and apolipoprotein B (apoB) reductions but minimal weight change. Semaglutide achieved dose-dependent histologic and metabolic benefits, culminating in dual-endpoint improvement in the phase 3 ESSENCE trial. Liraglutide improved resolution in a small trial, while tirzepatide achieved both endpoints with large, dose-related weight loss. Non-invasive biomarkers paralleled histology, and adverse events were predominantly mild gastrointestinal effects. GRADE assessments indicated low certainty for between-class differences in histologic outcomes, high certainty favoring incretin therapies for weight reduction, and moderate certainty favoring resmetirom for lipid lowering. In conclusion, resmetirom, semaglutide, and tirzepatide each demonstrate clinically meaningful efficacy and tolerability in non-cirrhotic MASH, with distinct metabolic profiles, resmetirom as a lipid-centric, weight-neutral therapy and incretins as weight-centric, pleiotropic agents. Further direct-comparison trials are warranted to clarify their relative benefits on histology and cardiometabolic outcomes.

## Introduction and background

Metabolic dysfunction-associated steatotic liver disease (MASLD) has supplanted nonalcoholic fatty liver disease (NAFLD) as the preferred overarching term, while metabolic dysfunction-associated steatohepatitis (MASH) has replaced nonalcoholic steatohepatitis (NASH) to emphasize the pivotal role of cardiometabolic factors and to standardize research and clinical protocols [1,2]. MASLD affects approximately one in four people globally and is closely associated with obesity, insulin resistance, and type 2 diabetes, increasing the risks of cirrhosis, hepatocellular carcinoma, and cardiovascular events [1,3,4]. Current European and American guidelines prioritize risk-stratified case-finding in primary care, the application of non-invasive tests (NITs) to identify patients with likely F2-F3 fibrosis, and the prompt treatment of high-risk conditions in conjunction with thorough cardiometabolic optimization [3,4]. For regulatory reasons, biopsy-anchored surrogate endpoints, (i) remission of steatohepatitis without worsening of fibrosis, and (ii) ≥1-stage improvement in fibrosis without worsening of steatohepatitis, are recognized criteria for accelerated approval in non-cirrhotic disease [5].

Two therapeutic platforms presently predominate in late-phase development and clinical use. First, selective thyroid hormone receptor-β (THR-β) agonism with resmetirom, which specifically targets hepatic lipid metabolism and atherogenic dyslipidemia, has demonstrated substantial improvements in histologic outcomes at 52 weeks in a phase 3 biopsy cohort, accompanied by consistent reductions in low-density lipoprotein cholesterol (LDL-C) and apolipoprotein B, as well as a tolerability profile conducive to prolonged treatment [6-8]. Second, incretin-based therapies primarily act through weight reduction and metabolic improvements: subcutaneous semaglutide demonstrated improved histologic resolution of steatohepatitis in phase 2 NASH, but did not significantly improve fibrosis at 72 weeks [9], whereas the phase 3 ESSENCE interim analysis indicated substantial benefits for both resolution and fibrosis improvement at the obesity dose of 2.4 mg weekly [10]. Earlier evidence, including liraglutide, demonstrated improved resolution and reduced fibrosis progression in a small phase 2 trial [11,12].

Interpreting these signals requires attention to surrogate and mechanistic domains that complement histology. MRI-proton density fat fraction (MRI-PDFF) monitors hepatic steatosis and pharmacodynamic response; iron-corrected T1 (cT1) and magnetic resonance elastography provide information on disease activity and fibrosis that correlates with histopathology and may support treat-to-target strategies in clinical trials and practice [13]. Lifestyle-induced weight loss of ≥10% is strongly associated with the resolution of steatohepatitis and regression of fibrosis, underscoring the importance of considering weight change as an effect modifier when evaluating drug classes with distinct primary mechanisms, THR-β agonism (lipid-centric, weight-neutral) versus incretins (weight-centric, pleiotropic) [14]. Despite numerous high-quality placebo-controlled trials, no direct comparisons have been conducted between resmetirom and semaglutide or tirzepatide using standardized, biopsy-based outcomes. In light of recent guidelines advocating prompt identification and management of non-cirrhotic, biopsy-validated MASH, together with the distinct mechanistic profiles of these agents, a comprehensive biopsy-based evidence synthesis is needed to clarify comparative advantages in histology, imaging, atherogenic lipids, and safety, as well as to evaluate the certainty of evidence using Grading of Recommendations Assessment, Development and Evaluation (GRADE) [3-12].

The objective of this review is to compare resmetirom, semaglutide, and tirzepatide in adults with biopsy-confirmed, non-cirrhotic MASH (F2-F3) with respect to (a) MASH/NASH resolution without worsening of fibrosis and (b) improvement in fibrosis by at least one stage without worsening of steatohepatitis. Additionally, it aims to contextualize weight-adjusted effects, NIT responses (MRI-PDFF, cT1), lipid changes, and safety/tolerability, with the certainty of evidence evaluated using GRADE.

## Review

Methodology

Protocol and Registration

**Figure 1:**
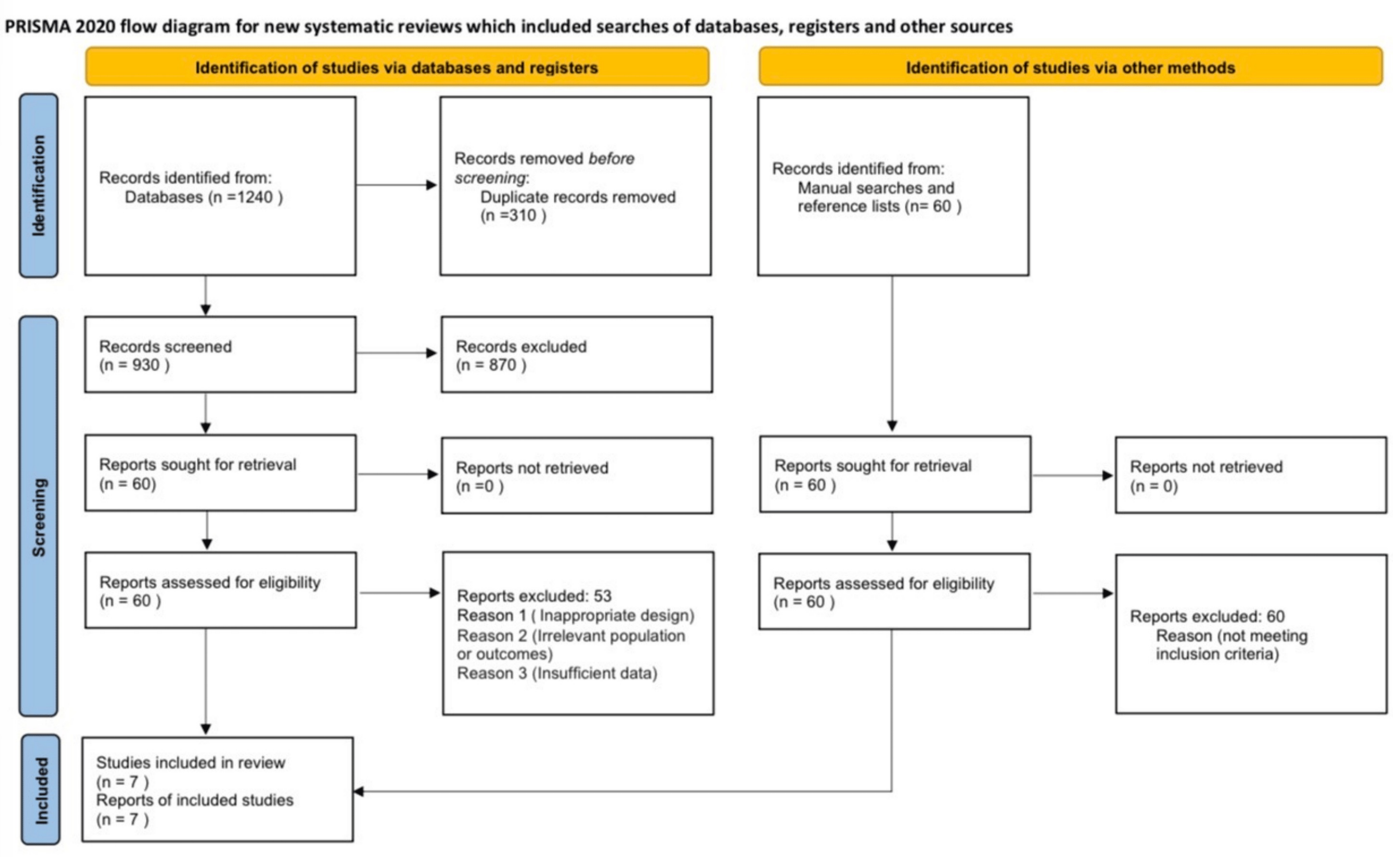
PRISMA 2020 flow diagram. PRISMA: Preferred Reporting Items for Systematic Reviews and Meta-Analyses.

This systematic review was conducted and reported in accordance with the PRISMA 2020 guidelines (Figure 1). The comprehensive protocol, including the eligibility criteria and data extraction methodology, was prospectively filed in PROSPERO (reg. no. CRD420251230032) before the commencement of data collection. All methods adhered to international standards for transparent and reproducible systematic reviews.

Eligibility Criteria

Eligible studies comprised randomized controlled trials (RCTs) including individuals aged 18 years or older with biopsy-confirmed, non-cirrhotic MASH/NASH, primarily at fibrosis stages F2-F3. The interventions of interest included resmetirom (a THR-β agonist), GLP-1 receptor agonists (semaglutide, liraglutide), and the dual GIP/GLP-1 receptor agonist tirzepatide. To ensure comparability, included studies were required to report at least one of the two regulatory biopsy-based surrogate endpoints recognized by the US FDA for non-cirrhotic MASH: (1) histologic resolution of steatohepatitis without worsening of fibrosis, or (2) improvement in fibrosis by ≥1 stage without worsening of steatohepatitis. Exclusion criteria included non-randomized or uncontrolled designs, pediatric or cirrhotic populations, combination pharmacotherapy trials, and studies without histology data.

Information Sources and Search Strategy

A comprehensive search was conducted in PubMed (MEDLINE), Embase, and the Cochrane Central Register of Controlled Trials (CENTRAL) from October 2015 to October 2025. The search strategy integrated controlled vocabulary and free-text terms pertaining to disease and intervention concepts: (“NASH” OR “MASH” OR “NAFLD” OR “MASLD”) AND (“resmetirom” OR “MGL-3196” OR “semaglutide” OR “liraglutide” OR “tirzepatide” OR “GLP-1” OR “GIP/GLP-1”) AND (random* OR placebo OR “double-blind”). Further records were obtained from ClinicalTrials.gov, the WHO ICTRP, reference lists of relevant publications, and proceedings from the annual meetings of American Association for the Study of Liver Diseases (AASLD) and European Association for the Study of the Liver (EASL). Only full-text, peer-reviewed human RCTs published in English were considered.

Study Selection and Data Extraction

Two reviewers independently screened all retrieved citations and full texts, resolving disagreements through discussion or consultation with a third reviewer. Extracted data included study design, country, setting, randomization and blinding methods, sample size, fibrosis stage distribution, intervention and comparator details, duration of treatment and follow-up, biopsy methodology, primary and secondary endpoints, imaging or biochemical biomarkers, lipid and metabolic outcomes, and adverse events. The final dataset comprised seven trials: MAESTRO-NASH 2024, MGL-3196 (Lancet 2019), MAESTRO-NAFLD-1 2023, semaglutide NEJM 2021, semaglutide ESSENCE 2025, liraglutide LEAN 2016, and tirzepatide SYNERGY-NASH 2024. Data accuracy was verified through cross-checking with supplementary materials and clinical trial registries.

Outcomes of Interest

The primary outcomes were (1) MASH/NASH resolution without worsening of fibrosis and (2) fibrosis improvement by at least one stage without worsening of steatohepatitis, assessed at 48-72 weeks. Secondary outcomes included changes in body weight, non-invasive imaging metrics (MRI-PDFF, cT1), serum lipids (LDL-C, ApoB, triglycerides), liver enzymes (alanine aminotransferase (ALT), aspartate aminotransferase (AST), gamma-glutamyl transferase (GGT)), glycemic parameters (HbA1c where available), and safety variables, including total adverse events, serious adverse events, and adverse event-related discontinuations.

Risk of Bias and Certainty Assessment

Each trial was critically appraised using the Cochrane Risk of Bias 2.0 tool across the domains of randomization, deviations from intended interventions, missing outcome data, measurement of outcomes, and selective reporting. The certainty of evidence for each outcome was evaluated according to the GRADE framework. Evidence was downgraded where appropriate for risk of bias, inconsistency, indirectness, or imprecision. Results are summarized below representing pairwise comparisons among resmetirom, semaglutide, and tirzepatide.

Data Synthesis

Given the limited number of eligible studies and heterogeneity in populations, dosing, and follow-up durations, no quantitative meta-analysis was performed. Findings were synthesized qualitatively, focusing on the direction and magnitude of effects across trials. Evidence was interpreted narratively, highlighting consistency, clinical relevance, and weight-adjusted effects. Descriptive summary below present trial characteristics, histologic efficacy, metabolic and imaging outcomes, and safety data, complemented by GRADE certainty ratings.

This review analyzed data from previously published studies and publicly available trial reports; therefore, no ethical approval or patient consent was required.

Results

Study Selection and Characteristics

Seven randomized controlled studies satisfied the inclusion criteria, encompassing three pharmacological classes: THR-β agonists (resmetirom), GLP-1 receptor agonists (semaglutide, liraglutide), and dual GIP/GLP-1 receptor agonists (tirzepatide). Studies that appeared to satisfy the inclusion criteria but were available only as abstracts were excluded. The trials were global, with the exception of the resmetirom phase 2 and MAESTRO-NAFLD-1 safety/biomarker studies, which were conducted predominantly in the United States. Follow-up duration varied from 36 to 72 weeks, with biopsy-confirmed efficacy assessments conducted at 48 to 72 weeks where required. Dosing corresponded to licensure-relevant ranges: resmetirom 80/100 mg once daily; semaglutide 0.4 mg daily or 2.4 mg weekly; tirzepatide 5/10/15 mg weekly. Comprehensive design and demographic information, including site geographies, is summarized in Table 1.

**Table 1:** Study characteristics (design, population, and endpoints). Trial-level design and PICO features for all included randomized studies of resmetirom, GLP-1 receptor agonists, and dual GIP/GLP-1 agonists in biopsy-confirmed NASH/MASH, as well as the safety/biomarker RCT in presumed NAFLD. The first column lists the first author and publication year; the second column lists the investigational drug. “N (analyzed)” reflects the randomized or prespecified efficacy population used for the primary endpoints. “Countries” indicates the geographic distribution of randomized sites. Histology endpoints follow each trial’s prespecified definitions and assessment timing. DB-RCT: Double-blind randomized controlled trial; F1B-F3: Fibrosis stages (including bridging fibrosis, as specified); HFF: Hepatic fat fraction; ITT/mITT: Intention-to-treat/modified intention-to-treat; MASH: Metabolic dysfunction-associated steatohepatitis; MASLD: Metabolic dysfunction-associated steatotic liver disease; NAFLD/NASH: Nonalcoholic fatty liver disease/steatohepatitis; NITs: Non-invasive tests; PDFF: Proton density fat fraction; QD/QW: Once daily/once weekly; RCT: Randomized controlled trial; THR-β: Thyroid hormone receptor beta; VCTE: Vibration-controlled transient elastography; PICO: Population, Intervention, Comparator, Outcome; GLP-1: Glucagon-like peptide-1; GIP: Glucose-dependent insulinotropic polypeptide; NASH: Nonalcoholic steatohepatitis.

Author, year	Drug	Class	Phase / Design (dose and duration)	N (analyzed)*	Population / key inclusion	Histology endpoints	Imaging / Biomarkers	Metabolic endpoints	Countries
Harrison SA et al. (2024) [6]	Resmetirom (MGL-3196)	THR-β agonist	Phase 3, DB-RCT; 80 mg or 100 mg vs placebo; biopsy at baseline and 52 wk	966 (biopsy cohort for primary endpoints)	Biopsy-confirmed NASH; F1B-F3; at-risk NASH	Co-primary: (1) NASH resolution without fibrosis worsening; (2) ≥1-stage fibrosis improvement without NASH worsening (both at 52 wk)	LDL-C, apoB (24 wk)	Cardiometabolic labs (e.g., LDL-C)	Multinational (15 countries): United States, Australia, Austria, Belgium, Canada, France, Germany, Hungary, Israel, Italy, Mexico, Poland, Spain, Switzerland, United Kingdom
Harrison SA et al. (2019) [7]	Resmetirom (MGL-3196)	THR-β agonist	Phase 2, DB-RCT; 80 mg vs placebo; 36 wk	125 (ITT)	Biopsy-confirmed NASH; HFF ≥10%; F1-F3	Key secondary: NASH resolution without fibrosis worsening; ≥1-stage fibrosis improvement	Primary: MRI-PDFF change at 12 wk; also assessed at 36 wk	Lipids; liver enzymes	United States (multicenter: 25 centers, 18 active sites)
Harrison SA et al. (2023) [8]	Resmetirom	THR-β agonist	Phase 3 (MAESTRO-NAFLD-1), DB-RCT (80/100 mg vs placebo) plus open-label arm; 52 wk	1,143 (safety population); double-blind arms n=972	Adults with NAFLD and presumed NASH; ≥3 metabolic risk factors; included a special safety cohort	Safety-focused; no mandated paired biopsy	MRI-PDFF (16 and 52 wk); liver stiffness/FibroScan CAP (52 wk); lipids	LDL-C, apoB, TG	United States (multicenter)
Newsome PN et al. (2021) [9]	Semaglutide (once-daily 0.1/0.2/0.4 mg)	GLP-1 RA	Phase 2, DB-RCT; 72 wk; daily SC dosing	320 (randomized)	Biopsy-confirmed NASH; F1-F3	Primary: NASH resolution without fibrosis worsening (72 wk); confirmatory secondary: ≥1-stage fibrosis improvement without NASH worsening	-	Weight, glycemia	Multinational (16 countries; 143 sites)
Sanyal AJ et al. (2025) [10]	Semaglutide (2.4 mg weekly)	GLP-1 RA	Phase 3 (ESSENCE) Part 1, DB-RCT; 72 wk histology readout of first 800 randomized patients (2:1)	800 (Part 1 histology cohort)	Biopsy-confirmed MASH; F2-F3	Co-primary: MASH resolution without fibrosis worsening; ≥1-stage fibrosis improvement without steatohepatitis worsening (72 wk)	NITs (e.g., VCTE, ELF, PRO-C3)	Weight and metabolic risk	International: 253 sites across 37 countries
Armstrong MJ et al. (2016) [11]	Liraglutide (1.8 mg daily)	GLP-1 RA	Phase 2, DB-RCT (LEAN); 48 wk	52	Biopsy-proven NASH	Primary: NASH resolution (48 wk)	-	Weight, glycemia	United Kingdom (multicenter; 4 centers)
Loomba R et al. (2024) [12]	Tirzepatide (5/10/15 mg weekly)	Dual GIP/GLP-1 RA	Phase 2, DB-RCT; 52 wk	190	Biopsy-confirmed MASH; F2-F3	Primary: MASH resolution without fibrosis worsening (52 wk); key secondary: ≥1-stage fibrosis improvement without MASH worsening	-	Weight, glycemia	130 sites in 10 countries: Belgium, France, Israel, Italy, Japan, Mexico, Poland, Spain, United Kingdom, United States

Histological Efficacy (Biopsy-Based Outcomes)

All key confirmatory trials across classes demonstrated superiority over placebo for at least one biopsy-based outcome, with variations in the pattern and magnitude of response. In MAESTRO-NASH, resmetirom improved the resolution of steatohepatitis without worsening fibrosis and improved fibrosis by at least one stage compared with placebo after 52 weeks. The resmetirom phase 2 study indicated directional benefit (primary MRI-PDFF response; biopsy subgroup findings were consistent); however, MAESTRO-NAFLD-1 did not require paired biopsy and instead adds biomarker context. Among incretin therapies, daily semaglutide (0.4 mg) improved histologic resolution at 72 weeks without a significant between-group effect on fibrosis improvement, whereas the ESSENCE part 1 results (2.4 mg weekly) showed significant benefits for both resolution and fibrosis improvement at 72 weeks. Liraglutide (LEAN) improved resolution and reduced fibrosis progression in a small phase 2 trial. SYNERGY-NASH (tirzepatide) demonstrated markedly superior resolution and a higher frequency of fibrosis improvement across doses at 52 weeks. Quantitative trial-level results and key observations are presented in Table 2.

**Table 2:** Key efficacy outcomes (primary and selected secondary outcomes). Biopsy-based categorical response rates are shown as percentages for the active and placebo groups for each study’s primary or confirmatory histology endpoints: (a) NASH/MASH resolution without fibrosis worsening, and (b) ≥1-stage fibrosis improvement without worsening of steatohepatitis at the protocol-specified time point. Where reported, an exploratory composite endpoint (“both achieved”) is also listed. Other clinically relevant signals (e.g., LDL-C change and enzyme improvements) are summarized to provide context for mechanism and class effects. Percentages are based on each trial’s prespecified efficacy population and imputation rules, typically non-responder imputation for missing biopsies. ALT/AST/GGT: Alanine aminotransferase/Aspartate aminotransferase/Gamma-glutamyl transferase; HbA1c: Glycated hemoglobin; LDL-C: Low-density lipoprotein cholesterol; NAS: NAFLD Activity Score; PBO: Placebo; MASH, metabolic dysfunction–associated steatohepatitis; VCTE: Vibration-controlled transient elastography; NASH: Nonalcoholic steatohepatitis; GLP-1: Glucagon-like peptide-1.

First author, year	Drug	Primary histology outcomes (vs placebo)	Other clinically important outcomes
Harrison SA et al. (2024) [6]	Resmetirom	NASH resolution (52 wk): 25.9% (80 mg) and 29.9% (100 mg) vs 9.7%. ≥1-stage fibrosis improvement: 24.2% and 25.9% vs 14.2%.	LDL-C↓ at 24 wk (both doses); apoB↓; effects were consistent across subgroups.
Harrison SA et al. (2019) [7]	Resmetirom	MRI-PDFF ≥30% responders (12 wk): 60.3% vs 18.4%. Biopsy subset (36 wk): NASH resolution 24.7% vs 6.5% (without fibrosis worsening).	ALT, AST, and GGT improved (especially with higher exposure); fibrosis responders (≥1-stage improvement) were 28.8% vs 23.5% (not statistically significant overall).
Harrison SA et al. (2023) [8]	Resmetirom	Safety-oriented trial; no paired-biopsy primary endpoint.	MRI-PDFF: -34.9% to -38.6% (16 wk); -28.8% to -33.9% (52 wk); lipids improved (LDL-C, apoB, TG).
Newsome PN et al. (2021) [9]	Semaglutide	NASH resolution (72 wk): 59% at 0.4 mg vs 17% with placebo; fibrosis improvement was not significantly different vs placebo.	Weight loss and glycemic control improved; safety was consistent with the GLP-1 receptor agonist class.
Sanyal AJ et al. (2025) [10]	Semaglutide	MASH resolution (72 wk): 62.9% vs 34.3%. ≥1-stage fibrosis improvement: 36.8% vs 22.4%. Composite (both achieved): 32.7% vs 16.1%.	Improvements in VCTE, ELF, and PRO-C3 were supportive; these analyses were not adjusted for multiplicity.
Armstrong MJ et al. (2016) [11]	Liraglutide	NASH resolution (48 wk): increased vs placebo; fibrosis progression reduced.	Weight loss and glycemic benefits were consistent with the GLP-1 receptor agonist class.
Loomba R et al. (2024) [12]	Tirzepatide	MASH resolution (52 wk): higher across the 5/10/15 mg groups vs placebo; fibrosis improvement (≥1 stage) was more frequent with tirzepatide.	Marked weight loss; dose-related gastrointestinal adverse events (see Table 3).

Non-Invasive Imaging, Metabolic, and Lipid Responses

Non-invasive signals paralleled histologic patterns while also revealing mechanistic signatures. Resmetirom demonstrated sustained reductions in MRI-PDFF during phase 2 and in the MAESTRO-NAFLD-1 biomarker study, while also improving atherogenic lipids (LDL-C, apoB, triglycerides), with minimal effect on body weight, highlighting a weight-neutral, liver-centric mechanism. Incretin-based therapies produced substantial weight loss and glycemic improvement; the effects of semaglutide were dependent on dose and duration, while tirzepatide demonstrated marked weight reduction and improvements in reported non-invasive liver metrics. Comprehensive cross-trial summaries are presented in Table 2 (efficacy context) and Table 3 (safety/mechanistic signals).

**Table 3:** Safety and tolerability (treatment-emergent adverse events and notable signals). Summary of TEAEs, common adverse events (≥10%), discontinuations (overall and adverse event-related, where available), and SAEs. Mechanistic cardiometabolic and hepatic signals (e.g., LDL-C, apoB, MRI-PDFF, cT1/MRE, liver enzymes) are included to contrast class profiles, resmetirom’s lipid-lowering effects without weight loss versus incretin-mediated weight loss with hepatic improvements. Values reflect on-treatment periods at the protocol-specified assessment time points. AE: Adverse event; apoB: Apolipoprotein B; cT1: Iron-corrected T1 mapping; D/C: Treatment discontinuation; MRE: Magnetic resonance elastography; MRI-PDFF: MRI-proton density fat fraction; SAE: Serious adverse event; TEAE: Treatment-emergent adverse event; TG: triglycerides.

Author, year	Drug	Overall safety signal	Most common AEs (≥10%)	Discontinuations / SAEs	Any notable class / mechanistic issues
Harrison SA et al. (2024) [6]	Resmetirom	Generally well tolerated vs placebo.	GI: diarrhea, nausea (more common vs placebo).	Low and similar between groups; serious adverse events were also similar across groups.	Expected lipid-lowering profile; THR-β selectivity is intended to reduce cardiac and thyroid off-target effects seen with older THR agonists.
Harrison SA et al. (2019) [7]	Resmetirom	Well tolerated over 36 wk.	GI: mild diarrhea, nausea (more common than with placebo).	Few discontinuations; no unexpected safety findings.	No meaningful weight effect; lipid improvements were substantial, including reductions in apoC-III and triglycerides.
Harrison SA et al. (2023) [8]	Resmetirom	TEAEs occurred in approximately 82% to 88% across arms; the open-label arm showed a similar class profile.	GI: diarrhea, nausea at treatment initiation; laboratory shifts were consistent with class effects.	Discontinuations were low; the safety primary endpoint was met.	Supports tolerability over 52 weeks at 80/100 mg.
Newsome PN et al. (2021) [9]	Semaglutide	Expected GLP-1 receptor agonist safety profile.	GI: nausea, vomiting; elevated amylase/lipase; gallbladder-related events.	Low discontinuation; no new safety signals.	Daily dosing; substantial weight loss and glycemic benefits.
Sanyal AJ et al. (2025) [10]	Semaglutide	Consistent with the known 2.4 mg obesity-dose safety profile.	GI (72 wk): nausea 36.3%, diarrhea 26.9%, constipation 22.3%, vomiting 18.6% (vs 13.2%, 12.2%, 8.4%, and 5.6%, respectively, with placebo).	Discontinuation: 2.6% semaglutide vs 3.3% placebo; SAEs 13.4% in both groups; no new safety signals.	High dose attainment/adherence; 88% reached the 2.4 mg dose.
Armstrong MJ et al. (2016) [11]	Liraglutide	Typical GLP-1 receptor agonist tolerability profile.	GI symptoms were most common.	Low; manageable with dose titration.	Class warnings such as pancreatitis and gallbladder events were monitored; no emergent signal was reported.
Loomba R et al. (2024) [12]	Tirzepatide	Good tolerability; dose-related GI adverse events.	GI: nausea, diarrhea, decreased appetite, constipation, and weight loss; most were mild to moderate.	Low overall discontinuation; serious adverse events occurred in 6% of tirzepatide-treated participants and 6% of placebo-treated participants; discontinuation due to AEs was 4% in both groups.	Dual GIP/GLP-1 effects; robust weight loss may mediate part of the benefit.

Safety and Tolerability

All agents demonstrated acceptable tolerability profiles consistent with class expectations. Resmetirom was generally well tolerated, with gastrointestinal symptoms, particularly diarrhea and nausea, occurring more frequently than with placebo, while discontinuation rates remained low. GLP-1-based regimens showed common gastrointestinal adverse effects; in the 72-week semaglutide ESSENCE study, the incidences of nausea, diarrhea, constipation, and vomiting were higher than those with placebo but did not result in increased dropout rates. Tirzepatide was associated with dose-dependent gastrointestinal adverse effects, with a low overall discontinuation rate. Table 3 synthesizes comparative safety data and common adverse events (≥10%).

Risk of Bias

The risk-of-bias assessments for the pivotal studies were predominantly classified as low risk across domains, including randomization, allocation concealment, blinding, and outcome measurement. Concerns were identified where relevant, such as the small sample size and biopsy-subgroup analyses in the resmetirom phase 2 study, the inclusion of an open-label arm in MAESTRO-NAFLD-1, and dose-finding and sponsor-related considerations for tirzepatide. The trial-level RoB 2 assessments are detailed in Table 4.

**Table 4:** Risk of bias (Cochrane RoB 2, trial-level assessment). Trial-level RoB 2 judgments are presented for the following domains: randomization process, allocation concealment, deviations from intended interventions/blinding, blinding of outcome assessment, incomplete outcome data, selective reporting, and other bias (e.g., interim analyses, small biopsy subsets, sponsor influence). The overall judgment follows the domain-level assessments, with priority given to the histology endpoints. Where a study reports an interim histology readout or a biopsy subset, this is reflected as “some concerns.” RoB 2: Cochrane Risk of Bias 2; DB: Double-blind; IWR: Interactive web-based randomization; OL: Open-label; PRO: Patient-reported outcome.

First author, year	Drug	Randomization process	Allocation concealment	Blinding (participants/personnel)	Blinding (outcome assessors)	Incomplete outcome data	Selective reporting	Other bias	Overall
Harrison SA et al. (2024) [6]	Resmetirom	Low (central randomization across sites)	Low	Low (DB)	Low (central pathology reads)	Low	Low (prespecified endpoints)	Some concerns (industry funding)	Low
Harrison SA et al. (2019) [7]	Resmetirom	Low	Low	Low	Low	Some concerns (biopsy subset and smaller phase 2 sample)	Low	Some concerns (phase 2 trial; industry funding)	Some concerns
Harrison SA et al. (2023) [8]	Resmetirom	Low	Low	Low in double-blind arms	Low	Low (safety-focused)	Low	Some concerns (open-label arm present)	Some concerns
Newsome PN et al. (2021) [9]	Semaglutide	Low	Low	Low	Low	Low	Low	Some concerns (industry funding)	Low
Sanyal AJ et al. (2025) [10]	Semaglutide	Low (2:1 randomization)	Low	Low	Low (central reads)	Low (prespecified histology cohort)	Low	Some concerns (interim histology analysis and industry funding)	Some concerns
Armstrong MJ et al. (2016) [11]	Liraglutide	Low	Low	Low	Low	Low	Low	Low	Low
Loomba R et al. (2024) [12]	Tirzepatide	Low	Low	Low	Low	Low	Low	Some concerns (phase 2 dose-finding trial; industry funding)	Some concerns

Comparative Analysis (GRADE, Pairwise)

Due to the absence of head-to-head trials, we synthesized comparative certainty using indirect evidence. The certainty regarding histologic superiority between resmetirom and semaglutide was low because of indirectness and inconsistency. However, weight change significantly favored semaglutide with high certainty, while LDL-C/apoB reduction likely favored resmetirom with moderate certainty. Adverse event-related discontinuation showed no significant difference, with moderate certainty. Histologic comparisons between resmetirom and tirzepatide were also uncertain and of low certainty; weight favored tirzepatide with high certainty, whereas lipid outcomes likely favored resmetirom with moderate certainty, with no significant difference in adverse event-related discontinuation (moderate certainty). Histologic comparisons between semaglutide and tirzepatide also yielded uncertain results. Weight and MRI-PDFF likely favored tirzepatide, with high and moderate certainty, respectively. The effects on lipids were uncertain and of very low certainty. Adverse event-related discontinuation showed no significant difference, with moderate certainty. The judgments are presented in Tables 5-7.

**Table 5:** GRADE summary of findings, Drug A (resmetirom) vs Drug B (semaglutide). Comparative certainty for resmetirom versus semaglutide was derived indirectly from placebo-controlled RCTs. Biopsy-based endpoints were treated as primary. “Uncertain” indicates that the available indirect evidence does not support a reliable directional claim. AE: Adverse event; ApoB: Apolipoprotein B; H2H: Head-to-head; MRI-PDFF: MR-proton density fat fraction; PBO: Placebo; RCT: Randomized controlled trial.

Outcome (time point)	Direct head-to-head evidence?	Evidence base used for indirect comparison	Comparative effect (A vs B)	Certainty (GRADE)	Downgrades / Reasons
MASH/NASH resolution without fibrosis worsening (52-72 wk)	No	A: Harrison SA et al. (2024) [6]; B: Newsome PN et al. (2021) [9], Sanyal AJ et al. (2025) [10]	Uncertain (both show benefit vs placebo; relative advantage unclear)	Low	Serious indirectness (different time points and populations); inconsistency across semaglutide phase 2 vs phase 3 studies.
≥1-stage fibrosis improvement without worsening of steatohepatitis (52-72 wk)	No	A: Harrison SA et al. (2024) [6]; B: Newsome PN et al. (2021) [9], Sanyal AJ et al. (2025) [10]	Uncertain (both show benefit vs placebo)	Low	Indirectness (no head-to-head comparison; different follow-up durations) plus inconsistency (phase 2 semaglutide neutral vs phase 3 positive).
Weight change (%) (52-72 wk)	No	A: Harrison SA et al. (2024) [6], Harrison SA et al. (2019) [7] (weight-neutral); B: Newsome PN et al. (2021) [9], Sanyal AJ et al. (2025) [10] (robust reduction)	Favors Drug B (semaglutide)	High	Large, consistent difference across RCTs; objective outcome; minimal concern for bias despite indirectness.
MRI-PDFF (% change) (12-52 wk)	No	A: Harrison SA et al. (2019) [7], Harrison SA et al. (2023) [8]; B: limited imaging data/subsets	Uncertain	Low	Indirectness plus imprecision (uneven imaging reporting across semaglutide trials).
LDL-C / ApoB change (24-52 wk)	No	A: Harrison SA et al. (2023) [8], Harrison SA et al. (2024) [6] (robust LDL-C/ApoB reduction); B: limited lipid reporting	Probably favors Drug A (resmetirom)	Moderate	Downgraded for indirectness; effect is large and consistent for Drug A, but less consistently reported for Drug B.
AE-related discontinuation (on-treatment)	No	A: Harrison SA et al. (2023) [8], Harrison SA et al. (2024) [6]; B: Newsome PN et al. (2021) [9], Sanyal AJ et al. (2025) [10]	No important difference detected	Moderate	Imprecision for relatively infrequent events; otherwise low risk of bias and similar absolute rates.

**Table 6:** GRADE summary of findings, Drug A (resmetirom) vs Drug C (tirzepatide). Comparative certainty for resmetirom versus tirzepatide is indirect. Weight loss consistently favors tirzepatide, while LDL-centric lipid outcomes consistently favor resmetirom; histology comparisons remain uncertain in the absence of head-to-head trials or a connected network meta-analysis. AE: Adverse event; ApoB: Apolipoprotein B; H2H: Head-to-head; MRI-PDFF: MR-proton density fat fraction; PBO: Placebo; RCT: Randomized controlled trial.

Outcome (time point)	Direct head-to-head evidence?	Evidence base used for indirect comparison	Comparative effect (A vs C)	Certainty (GRADE)	Downgrades / Reasons
MASH/NASH resolution without fibrosis worsening (52 wk)	No	A: Harrison SA et al. (2024) [6]; C: Loomba R et al. (2024) [12]	Uncertain (both superior to placebo)	Low	Indirectness (no head-to-head trial), imprecision (Drug C based on a single phase 2 trial), and differences in trial context/dose structure.
≥1-stage fibrosis improvement without worsening of steatohepatitis (52 wk)	No	A: Harrison SA et al. (2024) [6]; C: Loomba R et al. (2024) [12]	Uncertain (both beneficial vs placebo)	Low	Indirectness plus imprecision (Drug C based on a single phase 2 trial).
Weight change (%) (52 wk)	No	A: Harrison SA et al. (2024) [6], Harrison SA et al. (2019) [7] (weight-neutral); C: Loomba R et al. (2024) [12] (large reduction)	Favors Drug C (tirzepatide)	High	Large, consistent difference; objective outcome; robust magnitude for Drug C.
MRI-PDFF (% change) (12-52 wk)	No	A: Harrison SA et al. (2019) [7], Harrison SA et al. (2023) [8]; C: limited direct imaging data from Loomba R et al. (2024) [12]	Uncertain	Low	Indirectness, imprecision, and uneven imaging reporting across trials.
LDL-C / ApoB change (24-52 wk)	No	A: Harrison SA et al. (2023) [8], Harrison SA et al. (2024) [6] (robust reduction in LDL-C/ApoB); C: Loomba R et al. (2024) [12] (less prominent lipid effect reporting)	Probably favors Drug A (resmetirom)	Moderate	Downgraded for indirectness; consistency and magnitude favor Drug A across trials.
AE-related discontinuation (on-treatment)	No	A: Harrison SA et al. (2023) [8], Harrison SA et al. (2024) [6]; C: Loomba R et al. (2024) [12]	No important difference detected	Moderate	Imprecision for relatively infrequent events; overall similar discontinuation profiles.

**Table 7:** GRADE summary of findings, Drug B (semaglutide) vs Drug C (tirzepatide). Comparative certainty for semaglutide versus tirzepatide is indirect. Weight loss and fat-fraction surrogate outcomes probably favor tirzepatide, whereas histology comparisons remain uncertain in the absence of head-to-head data. Lipid effects are insufficient to determine a comparative advantage. AE: Adverse event; ApoB: Apolipoprotein B; H2H: Head-to-head; MRI-PDFF: MR-proton density fat fraction; PBO: Placebo; RCT: Randomized controlled trial; MASH, metabolic dysfunction–associated steatohepatitis; NASH: Nonalcoholic steatohepatitis; PDFF: Proton density fat fraction.

Outcome (time point)	Direct head-to-head evidence?	Evidence base used for indirect comparison	Comparative effect (B vs C)	Certainty (GRADE)	Downgrades / Reasons
MASH/NASH resolution without fibrosis worsening (52-72 wk)	No	B: Newsome PN et al. (2021) [9], Sanyal AJ et al. (2025) [10]; C: Loomba R et al. (2024) [12]	Uncertain (both superior to placebo)	Low	Indirectness (no head-to-head trial; different follow-up durations) plus imprecision (Drug C based on a single phase 2 trial).
≥1-stage fibrosis improvement without worsening of steatohepatitis (52-72 wk)	No	B: Sanyal AJ et al. (2025) [10], with Newsome PN et al. (2021) [9] showing no significant difference; C: Loomba R et al. (2024) [12]	Uncertain	Low	Indirectness plus inconsistency across Drug B trials and imprecision for Drug C.
Weight change (%) (52-72 wk)	No	B: Newsome PN et al. (2021) [9], Sanyal AJ et al. (2025) [10]; C: Loomba R et al. (2024) [12]	Favors Drug C (tirzepatide)	High	Large, consistent difference across trials; objective outcome; robust magnitude.
MRI-PDFF (% change) (52 wk)	No	B: limited imaging data/subsets; C: limited direct imaging data in the included MASH RCT	Uncertain	Low	Indirectness, imprecision, and sparse/uneven imaging reporting across both Drug B and Drug C trial sets.
LDL-C / ApoB change (24-72 wk)	No	B: limited/lower-magnitude lipid effects; C: modest lipid effects	Uncertain / no important difference	Very low	Indirectness, imprecision, and sparse/heterogeneous lipid reporting for Drugs B and C in MASH RCTs.
AE-related discontinuation (on-treatment)	No	B: Newsome PN et al. (2021) [9], Sanyal AJ et al. (2025) [10]; C: Loomba R et al. (2024) [12]	No important difference detected	Moderate	Imprecision for relatively infrequent events; overall similarly low discontinuation rates.

Comprehensive Synthesis

Collectively, biopsy-anchored trials support the efficacy of resmetirom, semaglutide (including the 2.4 mg weekly regimen), and tirzepatide versus placebo in non-cirrhotic, at-risk MASH, with each exhibiting a distinct mechanistic profile, resmetirom providing lipid-focused improvements alongside histologic benefits in a weight-neutral context, while incretin-based therapies produce weight-focused metabolic improvements with converging histologic signals at higher doses and longer durations. The comparative advantages for histology across classes remain uncertain in the absence of direct comparative data, but indirect comparisons suggest that weight loss favors incretins, whereas lipid reduction favors resmetirom. These findings, together with the predominantly low risk of bias, support the therapeutic relevance of all three pathways and define clear priorities for future direct-comparison trials.

Discussion

This systematic review consolidates findings from seven randomized trials evaluating three distinct drug classes for biopsy-confirmed, non-cirrhotic MASH/NASH: the THR-β agonist resmetirom, GLP-1 receptor agonists (liraglutide and semaglutide), and the dual GIP/GLP-1 agonist tirzepatide. In multiple studies, biopsy-derived efficacy indicators favored active therapy over placebo for NASH/MASH resolution and, to a lesser extent, for ≥1-stage fibrosis improvement, accompanied by class-specific cardiometabolic profiles: resmetirom resulted in substantial, consistent reductions in atherogenic lipids with neutral effects on body weight, while incretin-based therapies led to significant, dose-dependent weight loss alongside improvements in hepatic fat and aminotransferase levels. The trial-level trends are summarized in our characteristics table (Table 1), pooled efficacy signals (Table 2), and safety profiles (Table 3), and were assessed as showing a low-to-moderate risk of bias overall (Table 4).

The three GRADE Summary-of-Findings tables clearly illustrate that, at present, the comparative certainty between classes is low for biopsy endpoints but high for their differing effects on weight and lipids (Tables 5-7). Biopsy continues to be the regulatory gold standard and our principal anchor outcome; nevertheless, its interpretation and responsiveness have recognized limitations. The NAFLD Activity Score (NAS) and traditional Brunt grading/staging form the basis of endpoint definitions but were designed for descriptive pathology rather than as surrogates for medication response [15,16]. Sampling variability and reader discordance can attenuate treatment effects and widen confidence intervals, particularly when biopsy subgroups or missing-at-random assumptions are used in studies [17]. The issues evident in the imputation criteria and central reading strategies of several included trials support our decision to supplement histology with convergent non-invasive biomarkers in Table 2 and our certainty assessments in Tables 5-7 [15-17]. Our review adhered to current reporting and evaluation standards (PRISMA 2020, RoB 2, and GRADE), enhancing internal validity and clearly delineating the limitations of indirectness in cross-class comparisons [18-20].

Interpreting Class-Specific Benefit Profiles

Resmetirom demonstrates a distinct and consistent pharmacodynamic effect, evidenced by significant reductions in LDL-C and apoB, as well as histologic improvements in F2-F3 MASH, and it is currently incorporated into U.S. prescribing guidance for adults with non-cirrhotic NASH with moderate to advanced fibrosis [21]. Lowering atherogenic lipoproteins causally reduces ASCVD risk, providing a meaningful, weight-independent benefit on extrahepatic outcomes that complements hepatic histology [22-23]. Simultaneously, semaglutide 2.4 mg administered weekly has shown a 20% reduction in major adverse cardiovascular events (MACE) among individuals with established cardiovascular disease (CVD) and overweight or obesity, but without diabetes, indicating that weight-focused incretin therapy improves major outcomes at the population level. GLP-1 and dual GIP/GLP-1 agonists achieve substantial, sustained weight loss, the primary modifiable factor in NASH resolution, whereas resmetirom does not alter weight but reliably reduces hepatic fat and atherogenic lipids. Tirzepatide produced approximately 15% to 21% mean weight loss over 72 weeks in SURMOUNT-1, depending on dose [24], and in type 2 diabetes, the SURPASS-3 MRI substudy demonstrated significant reductions in liver fat using MRI-PDFF, accompanied by concurrent improvements in liver tissue health measures [25]. The mechanistic differences correspond clearly to our SoF tables: weight-related outcomes strongly favor incretins; LDL/apoB outcomes moderately favor resmetirom; and histologic comparisons across classes remain uncertain in the absence of head-to-head trials.

Role of Non-Invasive Tests (NITs) in Interpreting Treatment Effects

Numerous lines of evidence support MRI-based biomarkers as responsive surrogates that correlate with histologic response. A meta-analysis of individual patients and subsequent pooled studies have validated the superior diagnostic performance of MRE for assessing fibrosis in NAFLD and provided clinically applicable stiffness thresholds [25-27]. A relative reduction of ≥30% in MRI-PDFF independently correlates with NASH resolution and an improvement of ≥2 points in NAS across studies, establishing a credible early pharmacodynamic benchmark [27]. cT1 (iron-corrected T1) correlates with histologic activity and shows emerging associations with outcomes, positioning it as a useful complement to PDFF and stiffness for monitoring purposes [28,29]. These data guided our extraction of imaging outcomes (Table 2) and our GRADE assessments in cases with sparse or absent histology.

Clinical Implications

Resmetirom is an effective treatment for non-cirrhotic, fibrotic MASH (F2-F3), providing both histologic benefits and significant LDL/apoB lowering. This makes it an appealing option for patients with high cardiometabolic risk or those who are unable to tolerate weight-loss medications [21,22]. Incretin-based therapy is effective for weight loss, glycemic improvement, and hepatic fat reduction, and has been shown to provide cardiovascular benefit even in the absence of diabetes. Our synthesis suggests two complementary strategies for fibrosis regression: (i) lipid-centric THR-β agonism (resmetirom) for systemic atherogenic risk and hepatic histology, and (ii) weight-centric incretin therapy (semaglutide/tirzepatide) to maximize the probability of NASH resolution, recognizing that durable ≥10% weight loss is a strong driver of histologic improvement and that surgical benchmarks (e.g., bariatric surgery) set the upper bound for weight-mediated regression [30-35].

Combination and Sequence

Early randomized data suggest that combining semaglutide with drugs targeting FXR or ACC pathways (e.g., cilofexor/firsocostat) can combine weight loss with direct hepatic pathway modulation [30]. FGF21 analogues (efruxifermin, pegbelfermin) and pan-PPAR agonists (lanifibranor) have shown promising histologic and metabolic signals, making them potential partners in future regimens targeting steatosis, inflammation, and fibrosis [31-33].

Strengths and Limitations

Strengths include consistent directionality of effect against placebo across classes (Table 2), mechanistic coherence (Table 3), and a low-to-moderate risk of bias in trial design (Table 4). However, across-class comparisons in our SoF tables (5-7) are inherently indirect because of differences in populations, dosages, follow-up windows (52 vs 72 weeks), histologic assessment frameworks, and imaging techniques, which we downgraded accordingly [18-20,36-38]. Some trials relied on biopsy subgroups or interim histology reads, leading to uncertainty. Additionally, histology endpoints in compensated cirrhosis remain challenging (e.g., semaglutide did not show fibrosis benefit over 48 weeks in NASH cirrhosis) [39,40]. Until head-to-head comparisons or a linked network meta-analysis with robust transitivity are available, between-class differences in histology should be treated as hypothesis-generating rather than definitive [37,38].

Implications for Practice and Research

In routine care, a pragmatic “treat-to-target” approach could pair (i) a drug matched to the patient’s dominant risk phenotype (atherogenic dyslipidemia → resmetirom; obesity/diabetes → GLP-1/dual incretin) with (ii) a monitoring plan using validated NITs (e.g., PDFF for early fat response; cT1/MRE for activity/fibrosis risk) and (iii) durable lifestyle and weight-management strategies. For research, priorities include (1) head-to-head trials powered for histologic and clinical outcomes across classes; (2) mediation analyses to quantify weight-independent hepatic effects; (3) composite NIT-based endpoints calibrated to clinical outcomes; and (4) factorial or adaptive trials of rational combinations (e.g., THR-β agonist + incretin).

## Conclusions

This systematic analysis aggregates evidence from seven randomized trials demonstrating that resmetirom, semaglutide, and tirzepatide each produce significant histologic and metabolic improvements in people with non-cirrhotic MASH. All treatments showed an advantage over placebo for at least one biopsy-derived endpoint, accompanied by generally favorable safety profiles. Resmetirom is a lipid-focused, weight-neutral treatment that improves fibrosis and steatohepatitis while reducing LDL-C and apoB, thereby potentially mitigating the cardiometabolic risk associated with MASH.

Conversely, semaglutide and tirzepatide act as weight-focused, pleiotropic therapies that produce significant weight reduction, hepatic fat reduction, and metabolic improvements, with tirzepatide demonstrating the most pronounced overall effect. Comparative certainty across classes is low because the evidence is indirect; however, mechanistic complementarity suggests that combined or sequential approaches may yield optimal results. Future comparative and combination studies incorporating histologic, non-invasive, and cardiovascular endpoints are crucial to inform personalized, mechanism-driven therapy in MASH.
